# Microstructure of the Advanced Titanium Alloy VT8M-1 Subjected to Rotary Swaging

**DOI:** 10.3390/ma16216851

**Published:** 2023-10-25

**Authors:** Grigory S. Dyakonov, Tatyana V. Yakovleva, Sergei Y. Mironov, Andrey G. Stotskiy, Iulia M. Modina, Irina P. Semenova

**Affiliations:** 1 Laboratory of Multifunctional Materials, Ufa University of Science and Technology, 32 Zaki Validi st., 450076 Ufa, Russiamodina_yulia@mail.ru (I.M.M.);; 2 Laboratory of Mechanical Properties of Nanoscale Materials and Superalloys, Belgorod National Research University, 85 Pobeda str., 308015 Belgorod, Russia; s-72@mail.ru

**Keywords:** titanium alloy, rotary swaging, microstructure, electron backscatter diffraction (EBSD), ultrafine-grained structure, second-phase particles

## Abstract

In this study, the microstructural behavior of the advanced Ti-5.7Al-3.8Mo-1.2Zr-1.3Sn-0.15Si (VT8M-1) alloy during rotary swaging (RS) was investigated. VT8M-1 has increased heat resistance and is considered a replacement for the Ti-6Al-4V alloy. It was shown that, during RS, the evolution of the primary a phase is characterized by the formation of predominantly low-angle boundaries according to the mechanism of continuous dynamic recrystallization. The density of low-angle boundaries increases three times: from 0.38 µm^−1^ to 1.21 µm^−1^ after RS. The process of spheroidization of the lamellar (a + b) component is incomplete. The average size of globular a and b particles was 0.3 μm (TEM). It is shown that the microstructures after RS (ε = 1.56) and equal-channel angular pressing (ECAP) (ε = 1.4) are significantly different. The temperature–velocity regime and the predominance of shear deformations during ECAP contributed to a noticeable refinement of the primary a-phase and a more complete development of globularization of the lamellar (a+b) component. EBSD studies have shown that RS leads to the formation of a structure with a higher density of low- and high-angle boundaries compared to the structure after ECAP. The results are useful for predicting alloy microstructure in the production of long rods that are further used in forging operations.

## 1. Introduction

In the aviation industry and mechanical engineering, titanium alloys are used in parts for which low specific weight, high specific strength, and corrosion resistance are important [1]. For example, the use of titanium alloys in crucial structures instead of steel can reduce weight by up to 30%, which is critical for the aviation industry. With the development of aviation technology, the requirements for strength, fatigue, and heat-resistant properties of materials are constantly increasing. Therefore, the development of new alloys continues, and approaches are being developed that ensure the formation of special structures that provide an increased combination of properties.

The two-phase titanium alloy VT8M-1 (Ti-5.7Al-3.8Mo-1.2Zr-1.3Sn-0.15Si) is an advanced material that was recently developed for use at elevated temperatures instead of Ti-6Al-4V [2]. The operating temperature of the VT8M-1 alloy has an upper limit of ~450 °C, while the well-known Ti-6Al-4V is usually not used at temperatures above 350 °C. Thus, the use of the more heat-resistant alloy VT8M-1 will provide the ability of the parts to operate at higher temperatures, and under standard operating conditions, it will increase their service life. Therefore, the VT8M-1 alloy attracts the attention of developers and manufacturers of modern gas turbine engines.

Deformation and heat treatment are integral parts of the production cycle. The sequence of operations during deformation–heat treatment affects the structure, phase composition, and level of mechanical properties of parts made of titanium alloys. In a number of works aimed at transforming the initial coarse-grained structure into an ultrafine-grained (UFG) structure, the effectiveness of this approach for improving the physico-mechanical properties of alloys has been shown [3,4,5,6].

In this work, the RS method was used to produce rods. RS is an industrial high-performance deformation processing method whose principle is high-frequency deformation of the metal. During processing, the material is subjected to mixed stresses of biaxial compression and uniaxial tension [7]. The advantages of this method are high productivity, the possibility of obtaining long workpieces with increased mechanical properties, and a fine-grained structure [7,8,9]. It is noted that RS enables deformed metals with a small strain to be comparable in strength to those obtained through severe plastic deformation of metals with a large strain [7,10]. Processing materials using RS ensures the production of workpieces with both a gradient [11,12] and a relatively homogeneous structure [13,14,15,16]. The type of structure formed depends both on the material itself and on the conditions of deformation processing. Processing titanium and titanium alloys by RS allows for the obtaining of rods with an ultra-fine-grained structure, increased strength, good ductility, and fatigue properties [17,18,19].

RS of the titanium alloy Ti-6Al-4V is often used to produce seamless tubing [20,21] and ultra-fine-grained rods [13]. It has been shown that in the production of seamless tubing, RS leads to a reduction in grain sizes and the formation of a favorable radial texture from Ti-6Al-4V. Pipes with a radial texture have higher strength under biaxial load, better tensile properties, and better resistance to wall thinning [21]. RS of VT6 alloy at a temperature of 680–500 °C with a total strain of ε = 2.66 resulted in the formation of a homogeneous globular microstructure with a grain size of 0.4 μm [13]. The tensile strength of this material was 1315 MPa, and the elongation was 10.5%.

With regard to the VT8M-1 alloy, there are several studies in this direction [22,23,24,25]. In several works [22,23], ultrafine-grained workpieces with an average grain size of ~0.5 µm and a good combination of strength and ductility were obtained by equal-channel angular pressing (ECAP). EBSD studies of the microstructure during ECAP showed that spheroidization is controlled by the boundary-splitting mechanism [23]. Texture studies made it possible to analyze active slip systems during ECAP [23]. Textural features indicated the combined action of prismatic, basal, and pyramidal slip systems during the formation of the fine-grained alpha phase and the evolution of the primary globular alpha phase. In another work, the features of the microstructure (SEM, and TEM) and mechanical behavior of the VT8M-1 alloy after RS were discussed [24]. It has been shown that rotational swaging ensures the formation of a UFG structure and higher strength properties than after ECAP. It has been shown that during RS, silicide particles of the S2 type—(Ti,Zr)_6_Si_3_—are released [25]. An assessment of long-term strength showed that at operating temperatures of 300–400 °C, the UFG alloy demonstrates higher σ100 values, almost 100 MPa higher compared to the coarse-grained state. Thus, in previous works, the mechanical properties of UFG alloy VT8M-1 and the features of the fine structure were studied. However, the processes of fragmentation and evolution of grain boundaries in the VT8M-1 alloy during RS have not yet been characterized. Therefore, in this work, the EBSD method was chosen as the main method for studying the microstructure, which makes it possible to qualitatively and quantitatively study the parameters of the microstructure. In addition to this, microstructural analysis techniques such as SEM, TEM and EDS were also used.

## 2. Materials and Methods

**Figure 1:**
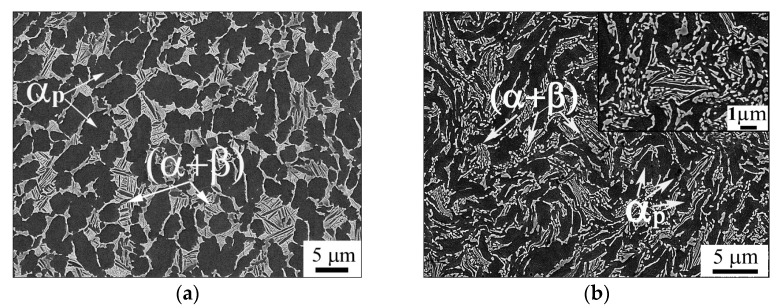
SEM micrographs showing (**a**) initial microstructure and (**b**) the microstructure produced after RS.

A rod of the VT8M-1 alloy (Ti-5.7Al-3.8Mo-1.2Zr-1.3Sn-0.15Si in wt.%) manufactured by VSMPO-AVISMA (Verkhnyaya Salda, Russia) was used as the research material. The as-received material had hot-rolled rods with a diameter of 70 mm, and the temperature of the β-transus was 980 ± 5 °C. The microstructure of the initial rod was of the duplex type (Figure 1a).

The deformation processing of the VT8M-1 alloy, which resulted in the formation of an ultrafine-grained structure, was carried out by RS with a stepwise reduction in diameter from 70 to 32 mm (at a strain rate of more than 300 mm/s). As a result, RS produced rods 4 m long. Several samples were cut from different parts of the rod for research. Before RS, the VT8M-1 alloy rod was heated in a furnace to 750 °C for 40 min. The equivalent strain was calculated according to the relation:

ε = ln(S_0_/S_1_) (1)

where S_0_ and S_1_ are the cross-sectional areas of the billet before and after deformation, respectively. 

The calculated equivalent strain after RS was ε = 1.56. RS being an industrial, inexpensive and high-performance method enables it to form an UFG structure in long-length rod billets [7]. The principle of RS is high-frequency deformation of the metal by anvils and the creation of centrosymmetric deformations.

In addition to the results of RS, this paper presents some data on the ECAP processing of the VT8M-1 alloy [22,23]. The billets were subjected to 2 and 4 ECAP passes using an angle within the ECAP die of ϕ = 120° with a temperature of 750 °C via route B_C_, in which the billet is rotated by 90° in a clockwise sense between consecutive passes. The workpiece was deformed at a rate of 4 mm/s; the equivalent degree of deformation per pass was ε = 0.7. The ECAP facility used in this work was equipped with a die heated to a temperature of 550 °C in order to provide isothermal conditions for billet straining. Taking into account the relatively fast ECAP deformation (4 mm/s) and the deformation heating of the workpiece, it is assumed that the temperature of the workpiece during the ECAP process can be close to 700 °C.

The microstructure was characterized by scanning electron microscopy (SEM) using JSM 6390 (JEOL, Tokyo, Japan) and by TEM using JEM-2100 (JEOL, Tokyo, Japan). The volume fraction of the primary α phase was determined from microstructure images (SEM) in accordance with ASTM E562 [26]. The elemental composition of the alloy was analyzed using a JEOL JEM 2100 microscope (JEOL, Tokyo, Japan) equipped with an EDS spectrometer. TEM samples were cut using electrical discharge machining, then mechanically thinned to a thickness of 100 µm, and electropolished on a TenuPol-5 machine with a solution of 5% perchloric acid, 35% butanol, and 60% methanol at a polishing temperature ranging from −20 to −35 °C.

The EBSD samples were prepared using conventional metallographic techniques, followed by a 24 h vibratory polishing with a colloidal silica suspension. EBSD was performed using a Hitachi S-4300SE (Hitachi, Japan) field emission scanning electron microscope operating at 25 kV and equipped with a TSL OIM EBSD system. A tolerance of 15° has been applied to distinguish low-angle boundaries (LABs) from high-angle boundaries (HABs). Grain-boundary density was calculated as a ratio of the total grain-boundary length on an EBSD map to the total area of the map.

## 3. Results

### 3.1. Microstructure Morphology

**Figure 2:**
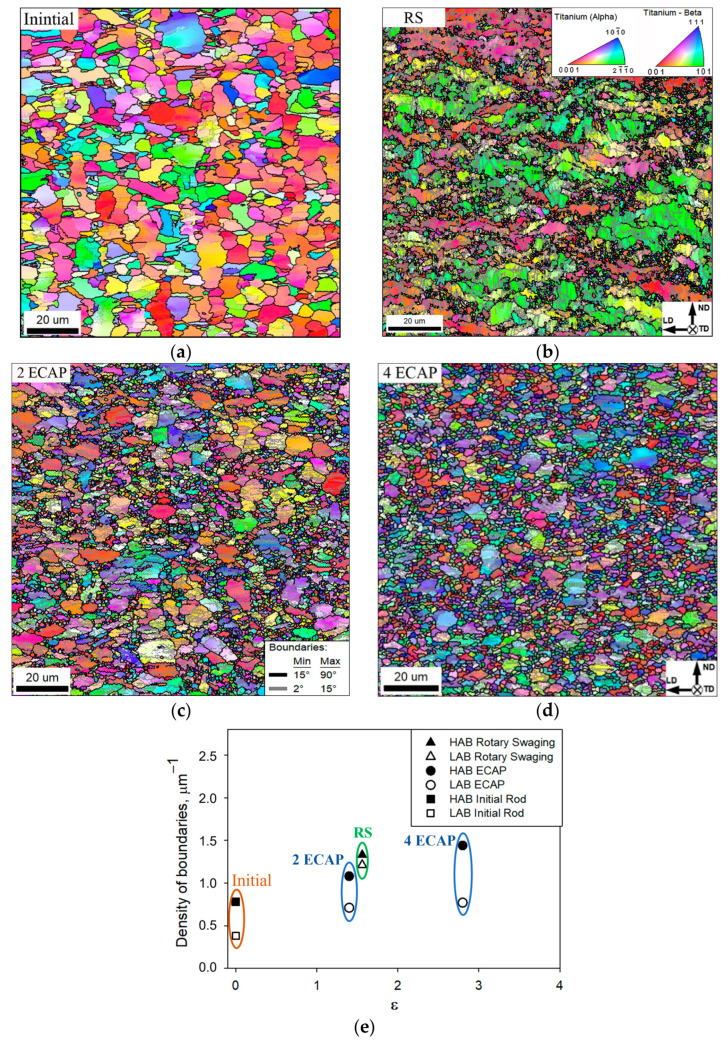
Selected portions of inverse-pole-figure (IPF) EBSD maps taken from (**a**) initial material, (**b**) material after RS (ε = 1.56), (**c**) material after 2 ECAP passes (ε = 1.4), (**d**) material after 4 ECAP passes (ε = 2.8), and (**e**) evolution of grain-boundary density as a function of cumulative strain. Note: the individual grains of α and β phases in the EBSD maps are colored according to their crystallographic orientation relative to the longitudinal axis of a processed billet; the color code triangles for α and β phases are shown in the top right corner of (**b**). HABs and LABs are depicted as black and gray lines, respectively. LD, ND and TD are longitudinal direction, normal direction, and transverse direction, respectively.

The initial duplex structure of the VT8M-1 alloy consists of a primary globular α phase (α_p_) and a+β transformed lamellar matrix (Figure 1a and Figure 2a). The fraction of the globular α phase and mean size of α particles were measured to be 68% and 4.5 μm, respectively. The β transformed matrix consisted of α and β laths about 0.2 µm thick (Figure 1a).

The RS led to a strong change in the morphology of the structure. The primary globular α phase was elongated in the direction of deformation, and most of the lamellar component was fragmented, resulting in the formation of small particles with a size of about 1 µm. The volume fraction of the primary α phase after RS remained almost unchanged at ~65%.

EBSD studies (here and hereafter, EBSD maps included both α and β phases; the individual EBSD maps for the b phase are given in Supplementary Figure S1) showed that RS led to the development of a bimodal structure with a high density of deformation-induced boundaries (Figure 2). From the EBSD map (Figure 2b), it was clear that fragmentation was actively developing in the lamellar α+β component. As a result, small equiaxed particles of α and β phases were formed, which are grouped between grains of the primary α phase. The proportion of low- and high-angle boundaries after RS was 49% and 51%, respectively (Table 1). Previously, to form an ultrafine-grained structure in the VT8M-1 alloy, the ECAP method was used [22,23]. Comparing the states after RS (Figure 2b) and after ECAP (Figure 2c,d), it is clearly seen that the morphologies of the microstructures differ significantly from each other. After ECAP processing, the primary α-phase grains retain their globular shape, unlike the structure after RS (Figure 2b–d). A rather large number of globular particles are observed around the primary α-phase grains (Figure 2c,d).

The evolution of the low- and high-angle boundaries was studied using a grain-boundary-density measure. After RS, the densities of LABs and HABs were comparable to each other, being 1.21 µm^−1^ and 1.33 µm^−1^, respectively (Figure 2e and Table 1). In contrast, two ECAP passes provided a completely different ratio of LABs and HABs. Specifically, the density of HABs was measured to be ~1.08 µm^−1^, while that of LABs was only ~0.71 µm^−1^. Four ECAP passes resulted in an increase of the density of HABs to 1.14 µm^−1^, while the density of LABs remained as low as ~0.77 µm^−1^. The predominance of HABs in the microstructure after 4 ECAP passes indicated a prevalence of the recrystallized microstructure. This suggestion was in line with the equiaxed microstructure morphology in this material condition (Figure 2d).

### 3.2. Grain Size Distribution

**Figure 3:**
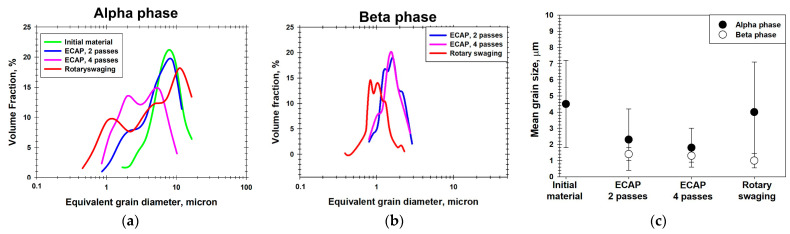
Effect of RS and ECAP on grain-size distributions in (**a**) α phase, (**b**) β phase, and (**c**) the mean grain size in both phases. Note: The grain-size statistics were derived from EBSD data.

The microstructure produced during RS was distinctly bimodal (Figure 2b), consisting of a combination of the relatively fine grains of the β phase (~1 μm) and the elongated grains of the primary α phase (Figure 3a,b).

The bimodal microstructure was also formed after two ECAP passes (Figure 2c). It consisted of the comparatively fine (~1.4 µm) β grains and the globular primary-α grains of 2.3 µm in diameter (Figure 3a,b). The further increase in ECAP strain to four passes led to a refinement of the α-grains to 1.8 μm (Figure 3a, Table 1). On the other hand, the mean size of the β grains remained nearly unchanged (Figure 3b, Table 1).

### 3.3. TEM Studies

**Figure 4:**
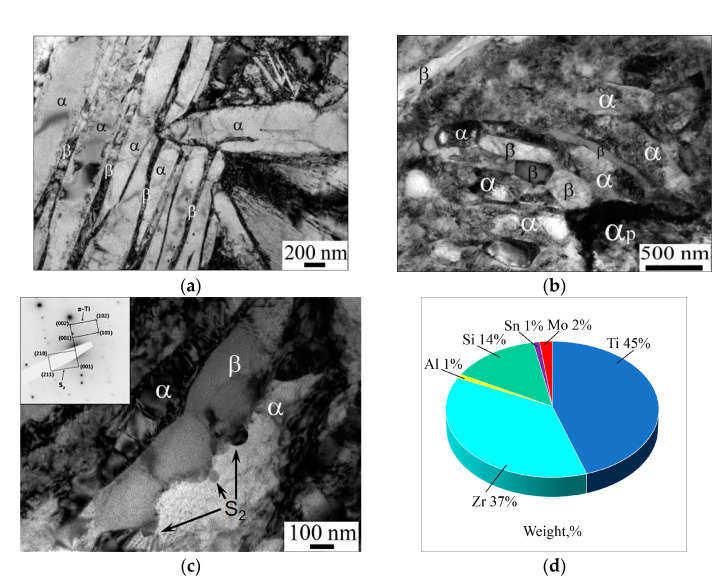
TEM micrographs showing (**a**) transformed-β microstructure of the initial material, (**b**) the typical microstructure produced during RS, (**c**) the nano-scale dispersoids in the heavily-deformed microstructure (arrowed), and (**d**) the averaged chemical composition of the dispersoids as revealed by EDS analysis. Note: The measured orientation relationship between the dispersoids and α phase is shown in the top left corner of (**c**).

TEM observations confirmed that the initial microstructure of the VT8M-1 alloy consisted of primary α grains and the alternating plates of α and β phases (Figure 4a). The RS resulted in the fragmentation of α and β plates (Figure 4b). The average size of globular α and β particles was 0.3 µm. On the other hand, it is important to emphasize that the fragmentation process was not completed and that a notable fraction of the severely deformed lamellar microstructure was still retained in the final material (Figure 4b). An important characteristic of the evolved microstructure was a high dislocation density in both the lamellar and globular constituents (Figure 4b).

From the scientific literature [27,28,29], it is known that in titanium alloys alloyed with Zr and Si, silicide particles are formed: (Ti,Zr)_5_Si_3_ (S1, c = 0.544 nm; a = 0.780 nm), (Ti,Zr)_6_Si_3_ (S2, c = 0.369 nm, a = 0.701 nm) and (Zr,Ti)_2_Si (S3). The nano-scale dispersoids of the (Ti, Zr)_6_Si_3_ phase may also be present in the VT8M-1 alloy [25]. Those were revealed in the present study (Figure 4c). The precipitates had a size of 80–100 nm and an elliptical shape, and their volume fraction was estimated to be 1–2%. The averaged chemical composition of the dispersoids as revealed by EDS analysis is shown in Figure 4d. Remarkably, the distinct patterns of grain-boundary bulging were revealed in the vicinity of the dispersoids (Figure 4c). This observation suggested an essential pinning effect exerted by the precipitates on the grain-boundary migration, thus being in line with a previous report in the scientific literature [30]. 

The formation of characteristic grooves at the α/β interface (Figure 4c) indicated the involvement of a specific mechanism for the transformation of the lamellar structure into a globular one, according to the boundary-splitting mechanism [31].

## 4. Discussion

**Figure 5:**
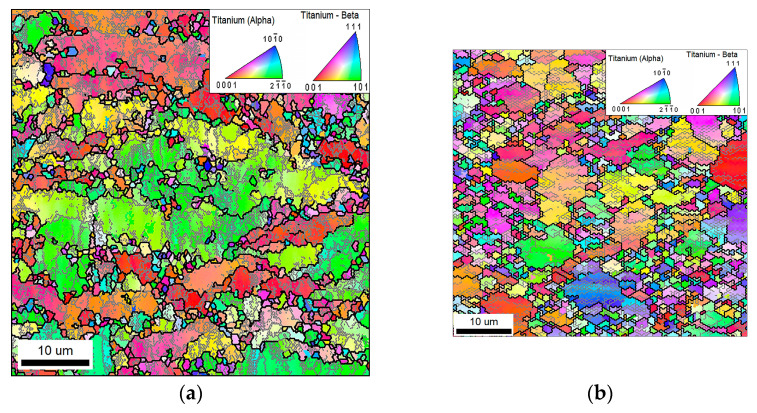
The selected portions of the EBSD orientation maps taken from the material subjected to the comparable strains using (**a**) RS (ε = 1.56) or (**b**) ECAP (ε = 1.4). Note: LABs and HAB are depicted as gray and black lines, respectively.

The present study showed that the microstructures produced during RS and ECAP were distinctly different from each other. This observation implied a difference in the underlying mechanisms of microstructural evolution. To get a closer inspection of this process in the case of RS and ECAP, the representative sections of EBSD maps were analyzed in Figure 5.

Given the relatively high LAB fraction in both material conditions, it is highly likely that the evolution of the primary globular α phase during RS and ECAP was governed by the continuous dynamic recrystallization. This mechanism implied a gradual increase in grain-boundary misorientation with strain and a subsequent gradual transformation of LABs into HABs [32]. In both cases, the LAB-to-HAB transformation process was far from being completed.

It is important to emphasize that the processed materials contained a relatively high dislocation density, as exemplified in Figure 4b. Thus, despite the microstructural changes in the present study being interpreted in terms of the continuous recrystallization, the final materials were not recrystallized in a classical sense. In fact, the microstructural evolution involved a redistribution of the lattice dislocations into the deformation-induced boundaries rather than the elimination of dislocations. Hence, the volume fraction of the recrystallized material could not be determined.

In spite of the fact that the material in both examined conditions experienced a comparable degree of cumulative strain of ~1.56, the evolved microstructures were distinctly different in terms of both microstructure morphology, and LAB and HAB fraction. 

The elongation of the primary α phase grains during RS was obviously associated with the severely-drawing of the processed billet. It is well visible that inside the elongated grains of the primary α phase, low-angle boundaries are formed that strive to intersect the grain across (Figure 5a). Some of these low-angle boundaries have already transformed into boundaries with a high-angle misorientation.

Unlike RS, after ECAP processing, the globular shape of the primary α phase is retained. This is promoted by the change in shear direction in the billet when route B_C_ is used and the absence of strong tensile stresses in the axial direction of the billet. ECAP is characterized by large shear strain components [3,33]. This contributes to the activation of additional slip systems (basal, prismatic, and pyramidal) and the more intensive formation of new deformation-induced boundaries [34]. However, intermediate heat treatment prior to each ECAP pass and a low strain rate (4 mm s^−1^) contribute to recovery. As a result, the alloy’s structure after ECAP processing is characterized by a relatively low density of low-angle boundaries (Figure 2c,e).

The results of microstructural studies indicate that the evolution of the alloy’s lamellar component and the formation of a fine-grained globular fraction of α and β particles during RS and ECAP processing were controlled by the same mechanism of groove formation and growth, but some features were revealed depending on the deformation method. 

Strong shear stresses provide efficient fragmentation of the alloy’s lamellar component during ECAP processing. With an increasing number of ECAP passes, the fragmented lamellar component becomes globularized grains. As shown by grain distribution by size, an increase in the number of ECAP passes has a beneficial effect on the alloy’s structure homogeneity (Figure 3c). It is necessary to point out that the ECAP deformation was accompanied by preliminary heat-treatment steps at T = 750 °C for 30 min prior to each pass. Such treatments contributed to the relaxation of internal stresses, rearrangement of the dislocation structure, formation of new globular particles, and perhaps some growth of the “old” spheroidized particles of the α and β phases. It is noted that under certain temperature conditions, globularization intensification may be provided due to the occurrence of the interphase recovery processes [32]. This statement is in good agreement with microstructural observations (Figure 2c,d). Most probably, this is the reason for the fact that, while the strain of ε ≈ 1.5 is commensurate, the mean size of the globular β particles after 2 ECAP passes is noticeably larger than that after RS (Table 1). It is also apparent that the fraction of the fine globular α and β particles in the alloy’s structure after ECAP processing is visibly larger as compared to the structure after RS (Figure 2b,c).

The RS involves high-speed deformation (more than 300 mm/s) compared to 4 mm/s for ECAP. During RS, biaxial compression and the uniaxial tensile stress state are implemented [7]. The absence of strong shear stresses during RS resulted in the fact that a part of the lamellar component was barely fragmented. As noted in several papers [34,35], globularization during monotonic deformation occurs in a non-uniform manner. This is related to the different orientation of the lamellae with respect to the acting stresses, their rotation in the direction of the “mild” orientations, and the subsequent geometrical reorientation along the direction of metal flow [36]. Analysis of the evolution of the lamellar component from the TEM images allows us to conclude that during the high-speed deformation by the RS, the spheroidization process via the formation and growth mechanism of grooves was not completed. This is clearly seen in Figure 4b,c. During RS, to ensure strain compatibility of the neighboring α and β phases, a high density of geometrically necessary dislocations develops in the lamellar component. The lamellae are fragmented by shear bands, resulting in the further formation of interphase boundaries that exit to the interphase surface. The next stage in the evolution of the lamellar structure is normally characterized by the growth of grooves on the α/β interphase surface and the formation of individual globular particles [37]. However, this process was not completed during RS.

It is important to point out that the quantification of a volume fraction of the globularized material in the present study was challenging due to the heavily deformed nature of the evolved microstructures. Hence, the standard approaches based on optical microscopy, SEM, or EBSD cannot be readily applied. This purpose can perhaps only be achieved by extensive TEM measurements. However, considering the relatively high labor intensity of this investigation, it is worthy of a separate study. At present, it can only be concluded that the extent of the globularization ability of RS was likely lower than that of ECAP. 

As demonstrated by the results of the present study, RS provides the formation of a specific ultrafine-grained structure. From a practical perspective, the formation of an ultrafine-grained structure will ensure enhanced strength properties, an increase in fatigue life, and design reliability. On the other hand, it is known that ultrafine-grained and nanostructured materials often exhibit reduced creep resistance due to high internal energy and a non-equilibrium state of boundaries [38].

In this work, an inhomogeneous UFG structure was obtained during RS. It represented a mixture of elongated fragmented grains of the primary α-phase and fine-grained particles of α- and β-phases. Previous studies of the long-term strength of the UFG VT8M-1 alloy showed that the formation of such a microstructure provides an increase in long-term strength. In particular, the 100 h creep resistance of the UFG alloy at 400 °C (close to the upper limit of the operating temperatures of the alloy) was 850 MPa compared to 750 MPa for the coarse-grained state [9]. Thus, an important task for the practical application of structural titanium alloys is the development of a microstructural design that provides the necessary set of mechanical properties. A separate problem, which will be solved in the future, is associated with the formation of a structure that, along with strength properties, will provide the required level of fracture toughness.

## 5. Conclusions

As a result of studying the microstructure of the VT8M-1 alloy using complementary SEM, EBSD, TEM, and EDS methods, the following was found:

1. During RS, a specific structure was formed, which consists of elongated grains of the primary α phase and ultrafine globular particles of α and β phases with an average size of 0.3 μm (TEM).
2. The evolution of the primary α-phase during RS was characterized by the formation of predominantly low-angle boundaries according to the mechanism of continuous dynamic recrystallization. This led to an increase in the proportion of low-angle boundaries from 32% in the initial state to 49% after RS. The density of low-angle boundaries increased three times, from 0.38 µm^−1^ in the initial state to 1.21 µm^−1^ after RS. According to EBSD analysis, as a result of RS, the mean size of the primary α phase grains has decreased from 4.5 μm to 4 μm.
3. Globularization of the lamellar α + β component was ensured by the boundary-splitting mechanism. The spheroidization process can be considered incomplete. Globularization of the lamellar component and the formation of new high-angle boundaries within the primary α-phase ensured an increase in the density of high-angle boundaries by almost two times, from 0.75 µm^−1^ in the initial state to 1.33 µm^−1^ after RS. The mean size of the globular particles of the β phase is 1 μm (EBSD-data).
4. It is shown that microstructures after RS and ECAP are significantly different. An amount of 2 ECAP passes (ε = 1.4) provided a reduction in the size of the primary alpha phase from 4.5 μm in the initial state to 2.3 μm after ECAP, while the RS treatment (ε = 1.56) resulted in a reduction of the primary alpha phase size to only 4 μm. The average particle size of the beta phase after RS was 1 μm (based on EBSD data), whereas the average beta particle size after two passes of ECAP was slightly larger and amounted to 1.4 μm.
5. It is shown that the processing of the VT8M-1 alloy by RS leads to the formation of a structure with a higher density of low- and high-angle boundaries as compared with the structure after ECAP processing. Such microstructural differences are caused by different kinetics of the development of continuous dynamic recrystallization and globularization processes.

## Figures and Tables

**Table 1:** Effect of RS and ECAP on microstructural characteristics (EBSD data).

Material Condition	Cumulative Strain, ε	LAB Fraction (%) LAB Density (µm^−1^)	HAB Fraction (%) HAB Density (µm^−1^)	Mean Size of α Grains (µm)	Mean Size of β Grains (µm)
Initial state	-	32% 0.38 µm^−1^	68% 0.75 µm^−1^	4.5	-
RS	1.56	49% 1.21 µm^−1^	51% 1.33 µm^−1^	4.0	1
2 ECAP passes	1.4	40% 0.71 µm^−1^	60% 1.08 µm^−1^	2.3	1.4
4 ECAP passes	2.8	40% 0.77 µm^−1^	60% 1.14 µm^−1^	1.8	1.3

## Data Availability

Not applicable.

## Supplementary Materials

The following supporting information can be downloaded at: https://www.mdpi.com/article/10.3390/ma16216851/s1. Figure S1: EBSD orientation maps of beta-phase taken from the (a) material after RS (ε = 1.56) and (b) material after 4 ECAP passes (ε = 2.8). The individual grains in the maps are colored according to their crystallographic orientation; the color code triangle is shown in the top right corner of (a).
