# Supplementary material for: Microstructure of the Advanced Titanium Alloy VT8M-1 Subjected to Rotary Swaging
Source: Materials (Basel). 2023 Oct 25;16(21):6851. doi: 10.3390/ma16216851 (PMC10650628; doi:10.3390/ma16216851)
Supplement: Supplementary file 1 [file materials-16-06851-s001.zip › materials-2595882-supplementary.pdf]

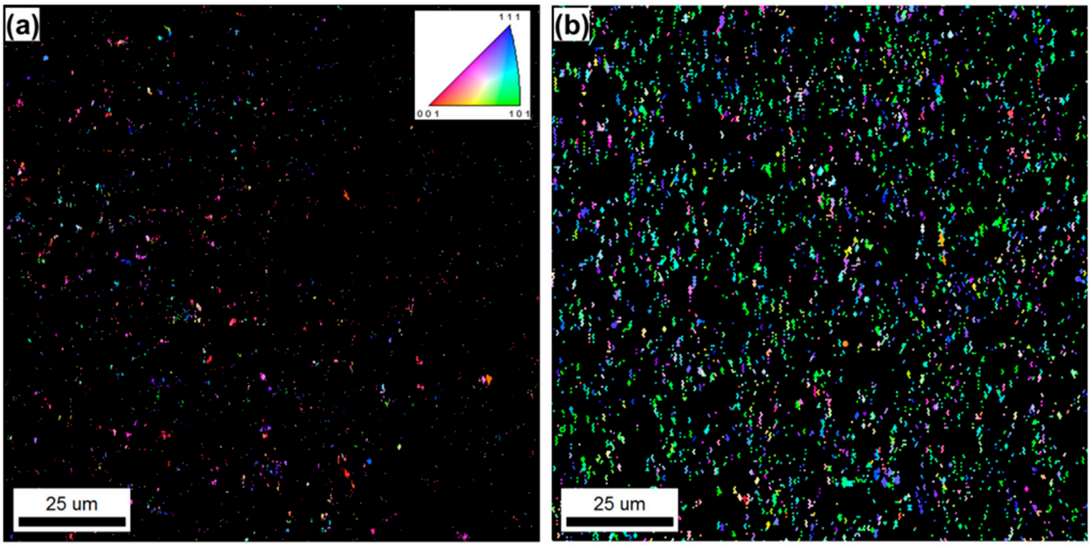

Supplementary Figure S1. EBSD orientation maps of beta-phase taken from the: (a) material after RS (ε=1.56) and (b) material after 4 ECAP passes (ε=2.8). The individual grains in the maps are colored according to their crystallographic orientation; the color code triangle is shown in the top right corner of (a).
